# Editorial: China and the world–Cultural differences, transmissions and transitions

**DOI:** 10.3389/fsoc.2022.979552

**Published:** 2022-08-15

**Authors:** Marc Oliver Rieger, Mei Wang, Yongjing Zhang

**Affiliations:** ^1^Department IV, University of Trier, Trier, Germany; ^2^WHU-Otto-Beisheim School of Management, Vallendar, Germany; ^3^GSPIA, Economics, University of Ottawa, Ottawa, ON, Canada

**Keywords:** China, culture, belt and road initiative (BRI), patriotism and nationalism, COVID-19, intellectual property rights (IPR), healthcare, economics

The role of China in the world has changed substantially in the past several decades: the “sleeping giant” has become an economic power. While China's rise lifted the majority of its population out of poverty and also helped other countries to harvest the fruits of globalization, this economic power has also led to increasing political and military controversies that have triggered international worries. Recent years have also brought back tighter control within China, putting pressure in particular on Chinese researchers and limiting their options for uncensored research.

How can we deal with this situation? An easy way out, which has indeed been seriously suggested by some Western China researchers, is to simply *not* conduct empirical research on China anymore: the argument is that if many questions cannot be asked in surveys, then research will be so limited that it is not worth pursuing. China research thus gets reduced to mainly reading government regulations and documents and trying to deduce reality from those. It should be clear by now that we do not agree with this view at all: empirical research is particularly pivotal in the current difficult times in order to understand what is really going on in China, and what Chinese people really think. Such research is difficult (it can even be framed as illegal “spying activity” by Chinese authorities) and we ought to admit its obvious limitations—e.g., sometimes people do not disclose their genuine thoughts owing to the sensitive nature of certain topics—even in an anonymous survey. Social science, however, has always been challenged by such restrictions: taboos exist even in completely open and liberal societies, yet researchers have never stopped researching them and trying to find clever ways to circumvent these hurdles. Good empirical research can do the same in China. The alternative would be to merely guess at what is going on in China and to publish opinions based on these conjectures—at a time where China research is particularly important. Speculations and viewpoints, however, should be left to opinion pieces in the media (and maybe to editorials like this). Academic research should always be based on facts and data, even if both are difficult to obtain.

We are excited that this special issue presents not only some examples of interesting and down-to-earth research on China, but also articles that collect such first-hand results to provide a better overview.

In “*The Impact of the ‘Belt and Road Initiative' on International Scholarship Students*,” Lea Shih and Wei Cao study empirically whether the Belt and Road Initiative (BRI) has a significant impact on international exchange. Using difference-in-differences methods, they found in particular that the number of Chinese government scholarships—one way to study in China—increased significantly in BRI countries as compared to non-BRI countries. The BRI is therefore valuable for cultural exchange, even if this might not have been its main purpose.

The article “*Uncritical Patriotism and Belief in COVID-19 Conspiracies*” by Rieger is based on surveys in Germany and China and investigates how uncritical (i.e., blind) patriotism affects belief in various conspiracy theories surrounding COVID-19. Since the origin of the pandemic in China is by now strongly denied by the Chinese government and the state-controlled propaganda, it was interesting to determine what Chinese people think about the origin of COVID-19 and whether patriotism plays a role in this. The article also analyses the difference between Chinese in Germany (who have access to a variety of media) and in China (with very limited access to non-Chinese media).

Since the West has also seen the destructive power of conspiracy narratives during the COVID-19 pandemic, but also more recently in relation to the war in Ukraine, this topic is not only interesting as a piece of research on China, but is relevant for other countries too. After all, conspiracy theories have flourished here as well: while Chinese narratives blame, e.g., the CIA for causing the disease, Western narratives blame, e.g., Bill Gates or the Chinese government. The above-mentioned article uncovers some of the underlying mechanisms that make people believe in such narratives.

Another China-related topic that has surfed the headlines for many years is intellectual property (IP) rights. The article “Intellectual Property Rights in China—A Literature Review on the Public's Perspective” aggregates research results that explore an unusual, but highly important question regarding IP: how do normal people perceive IP laws? What do they *know* about them? How do they think these laws *should* be? This perspective is obviously very important: On the one hand, law enforcement is difficult when people are unaware of infringement. On the other hand, if a large majority of people deem laws to be unjust, law-makers might have to consider possible adjustments. Although this latter point seems obvious, in particular in democratic countries, surprisingly little research is done on it. The article in question by Muehlfeld and Wang lays a foundation for further studies on this question (and potential comparisons with Western countries) by collecting previous articles that dealt with the perception of IP regulation in China.

Finally, the article “*About Face: How the People's Republic of China Harnessed Health to Leverage Soft Power on the World Stage*” by Paul Kadetz and Michael Stanley-Baker discusses an interesting excerpt from recent history in the interaction of China with the world, namely the perception of Chinese health programs under Mao Zedong by the West. Given that many of these programs were enforced in China during the Cultural Revolution (1966–1976), this article asks whether Western countries and organizations like the WHO “orientalized and romanticized” the healthcare measures of China.

This is not just a historic question, since the relation between international healthcare represented by the WHO and China is still complex today, as we have seen during the COVID-19 pandemic: here, the WHO was repeatedly criticized in Western media as being too uncritical about China, while in China, WHO reports were censored.

The articles in this special issue cover a broad range of topics that contribute to understanding China and the world in terms of cultural differences, transmissions and transitions.

## Author contributions

All authors listed have made a substantial, direct, and intellectual contribution to the work and approved it for publication.

## Conflict of interest

The authors declare that the research was conducted in the absence of any commercial or financial relationships that could be construed as a potential conflict of interest.

## Publisher's note

All claims expressed in this article are solely those of the authors and do not necessarily represent those of their affiliated organizations, or those of the publisher, the editors and the reviewers. Any product that may be evaluated in this article, or claim that may be made by its manufacturer, is not guaranteed or endorsed by the publisher.

